# A Case of Molar and Maxillary Absorption

**Published:** 1848-04

**Authors:** William A. Pease


### ARTICLE V.

### *A Case of Molar and Maxillary Absorption.* By William A. Pease.

[From the Dental Recorder.]

Some time in May last, a lady, Mrs. C——, called upon me and wished me to examine her face on the left side. To use her expression, "her cheek was falling in, constantly shrinking, and she did not know but it would continue so to do, till she was perfectly deformed." Upon examination, I found an indentation so large, as to be perfectly visible to any one in the room, somewhat resembling a very large dimple and not very dissimilar to a scar, when adhesion has taken place between the skin and periosteum, as is often witnessed in necrosis; though without a change of color. It was situated at the inferior edge of the os malar, at its junction with the os maxillary and near the posterior attachment of the masseter muscle, immediately above the antrum highmorianum. She complained of no pain, and no pressure, there was but slight tenderness. She said she had received no injury, nor experienced any pain or swelling of the part, and the first intimation she had of its presence, her attention was directed to it by a friend several weeks previous; that since that time, it had steadily, though slowly increased. There was a slight depression in the bone and less muscular and adipose matter than was natural above it. My attention was next directed to her mouth, which I found in a wretched condition; all the teeth in the left superior maxilla being defective, mostly broken off level with the alveolar, particularly the bicuspides; the first molaris, a very large tooth, was badly decayed, a mere shell and of a dark purple color; it having been filled several years since by a then respectable, self-styled dentist, with mineral paste; I think she said, he termed it succedaneum. It was then very dark, and oyxdized, and it was self-evident on the most superficial examination, that the juices of the mouth had free access to the whole, or nearly the whole surface of the filling. The second molaris was nearly broken away, as well as the dens sapientiæ and in a most filthy condition. The gums turgid, and slightly inflamed; bleeding on the slightest pressure, and of a dark, unhealthy color, though still retaining their attachment to the necks of the teeth. By gently striking the teeth with an instrument, there was a dull pain experienced immediately under the indentation on her cheek, and by tracing the external fangs of the teeth with the finger, there was a sensitiveness felt, particularly at their upper extremities. She was a strong, healthy woman; of a full habit, the sanguine temperament predominating; pulse full and quick; and she was judged to be about seven months advanced in pregnancy. This, combined with considerable dread of any operation, and a disquietude of nerves, together with an entire exemption from pain induced me to advise her to wait a short time; that I might watch the progress of the disease, and be better able to judge of its proximate cause, and the means most suitable to afford relief. About the first of September, she called again, said it was constantly increasing, though destitute of pain; that she believed the left side of her face would be badly deformed.

She was now less nervous; pulse strong and regular. She having in the interval accomplished the period of utero-gestation, and been delivered of a dead fœtus; said her attending physician had advised her to again consult me, and submit to any operation I might deem essential to her recovery; found a visible increase in the indentation, with considerable elongation, it being perpendicular, and much resembling an ellipsis, the lines of which had been produced to nearly a point. Upon again tapping the teeth with an instrument found increased sensitiveness, which had extended to the second molaris; with dull pain on pressure with the finger over the fangs; gums same as before. Advised the immediate removal of the teeth, if not for the sole, as a preliminary means of cure; founding my diagnosis on the fact, that the teeth were very large and strong, and would be likely to have corresponding long roots, which might perforate the plate of the antrum; being also dead, and extraneous substances, perhaps with ulcers attached to them, they might have lighted up an inflammation of the mucous membrane and periosteum, which extending to the bone, was producing its absorption; or the roots not extending to the antrum, being still foreign bodies, nature endeavoring to relieve herself of the burden, had induced a morbid, abnormal action, producing inflammation of the bone and process and a tendency to, if not caries. Though caries was not then judged to be present, from the absence of pain and much soreness and external swelling; neither was their apprehension of tumor or polypi. Extracted the second molaris, found the roots not as long as expected and unaccompanied with ulcers. The third next, as less trouble was anticipated from these than the first. On applying the forceps to the first, it immediately crushed in level with the alveolar, bringing away the large cement filling; divided the roots and extracted singly; they were quite long, but could not discover that they penetrated the sinus. She left, expecting to call in a few days: saw her after an absence of six weeks; the indentation on her face had not increased, it was free from pain or soreness, the gums healthy and naturally colored; the alveolar considerably absorbed and every thing indicating health. She expressed her conviction, that the disease was eradicated, which I judged to be the case.

Now, was this maxillary and malar absorption; for such I now consider it to have been; which had produced a lasting and unnecessary deformity, the result of diseased action induced by this large oxydated amalgam filling, or was there sufficient cause without it in the dead teeth and roots? To me it was evident that the tooth containing the filling was the most directly implicated in the disease, accompanied as it was by tenderness along, and at the extremity of its roots, at my first examination, and at the second, it had extended to the second molaris, thus showing if either had been the exciting cause, it must have been this one. But the question then will arise, was the disease, if from either, attributable to the filling or to the roots? If the former, why should it, after having remained in the mouth some five or six years without inconvenience, abundant time having elapsed for the complete evaporation of the mercury, at this late period produce these deleterious results? I am unwilling to charge it upon the fillings, yet am thoroughly convinced from the disease having been arrested by or spontaneously ceasing to progress simultaneously and with the extraction of the teeth, that there was some relation existing between them like cause and effect. Nothing like salivation or mercurial disease was witnessed and the whole appearance of the lady, at the time the teeth was extracted attested a healthy tone of the general system. The above treatment is all that I deem to have essentially to have effected the disease, no active remedial agent having been prescribed by me.

